# Effects of Hepatitis B Virus S Protein Exposure on Sperm Membrane Integrity and Functions

**DOI:** 10.1371/journal.pone.0033471

**Published:** 2012-03-28

**Authors:** XiangJin Kang, QingDong Xie, XiaoLing Zhou, FangZheng Li, JiHua Huang, DongLing Liu, TianHua Huang

**Affiliations:** Research Center for Reproductive Medicine, Shantou University Medical College, Shantou, China; Florida International University, United States of America

## Abstract

**Background:**

Hepatitis B is a public health problem worldwide. Viral infection can affect a man's fertility, but only scant information about the influence of hepatitis B virus (HBV) infection on sperm quality is available. The purpose of this study was to investigate the effect of hepatitis B virus S protein (HBs) on human sperm membrane integrity and functions.

**Methods/Principal Findings:**

Reactive oxygen species (ROS), lipid peroxidation (LP), total antioxidant capacity (TAC) and phosphatidylserine (PS) externalization were determined. The terminal deoxynucleotidyl transferase-mediated dUTP nick end labeling (TUNEL) assays and flow cytometric analyses were performed. (1) After 3 h incubation with 25 µg/ml of HBs, the average rates of ROS positive cells, annexin V–positive/propidium iodide (PI)-negative cells, Caspases-3,-8,-9 positive cells and TUNEL-positive cells were significantly increased in the test groups as compared to those in the control groups, while TAC level was decreased when compared with the control. The level of malondialdehyde (MDA) in the sperm cells exposed to 50 µg/ml of HBs for 3 h was significantly higher than that in the control (P<0.05–0.01). (2) HBs increased the MDA levels and the numbers of ROS positive cells, annexin V–positive/PI-negative cells, caspases-3, -8, -9 positive cells and TUNEL-positive cells in a dose-dependent manner. (3) HBs monoclonal antibody (MAb) and N-Acetylcysteine (NAC) reduced the number of ROS-positive sperm cells. (4) HBs decreased the TAC levels in sperm cells in a dose-dependent manner.

**Conclusion:**

HBs exposure could lead to ROS generation, lipid peroxidation, TAC reduction, PS externalization, activation of caspases, and DNA fragmentation, resulting in increased apoptosis of sperm cells and loss of sperm membrane integrity and causing sperm dysfunctions.

## Introduction

Hepatitis B is a public health problem worldwide. As estimated, two billion people have been infected with HBV [1]. The subviral particles of HBV are produced in vast excess during the life cycle of the virus, whose concentrations could reach 50–300 mg/ml in blood [2]. HBV is able not only to pass through the blood-testis barrier and enter the male germ cells but also integrate into their genomes [3]–[7].The previous work has confirmed that human sperm cells could serve as possible vectors for vertical transmission of HBV genes. After being introduced into the embryo via the sperm, HBV genes were replicated and expressed in the embryonic cells [7]–[10]. Furthermore, co-incubation of human spermatozoa with hepatitis B virus S protein, caused a significant loss of sperm mitochondrial membrane potential (MMP), reduced the sperm motility, and resulted in sperm death and diminished fertility [11]. However, the exact molecular mechanism of such events remains to be investigated.

Mitochondrial dysfunctions have been shown to increase production of ROS, which plays an important role in multiple cellular physiologic processes and in signaling processes [12], [13]. At low levels, ROS is necessary for normal functions of spermatozoa including capacitation, hyperactivation, motility, acrosome reaction, oocyte fusion and fertilization. In contrast, high levels of ROS can cause oxidative stress and induce pathophysiological changes in the spermatozoa [14], [15]. Human spermatozoa are particularly vulnerable to oxidative stress by virtue of lacking the cytoplasmic space to accommodate antioxidant enzymes, and the sperm plasma membrane contains lipids in the form of polyunsaturated fatty acids [16], [17]. In the presence of polyunsaturated fatty acids, ROS promotes a cascade of lipid peroxidation chain reactions, and ultimately leads to the production of cytotoxic aldehydes and affects membrane fluidity, mobility and fertilizing potential [18], [19]. ROS can also damage DNA by causing deletions, mutations, and other lethal genetic defects, which can lead to man's low fertility, higher rates of miscarriages and even increased incidence of morbidity in the offspring, including childhood cancers [20], [21].

Viral infection can actively elicit apoptosis, and higher proportion of apoptotic and necrotic sperm cells in the patients with chronic HBV infection has been documented [22]. Such phenomenon may be attributed to intrinsic and extrinsic factors such as toxin exposures and oxidative stress [23].

Thus, we assessed the oxidative stress and apoptotic features in sperm cells in the present study to further investigate the effects of HBs exposure on sperm membrane integrity and functions.

## Results

### ROS levels in sperm cells exposed to HBs

**Figure 1:**
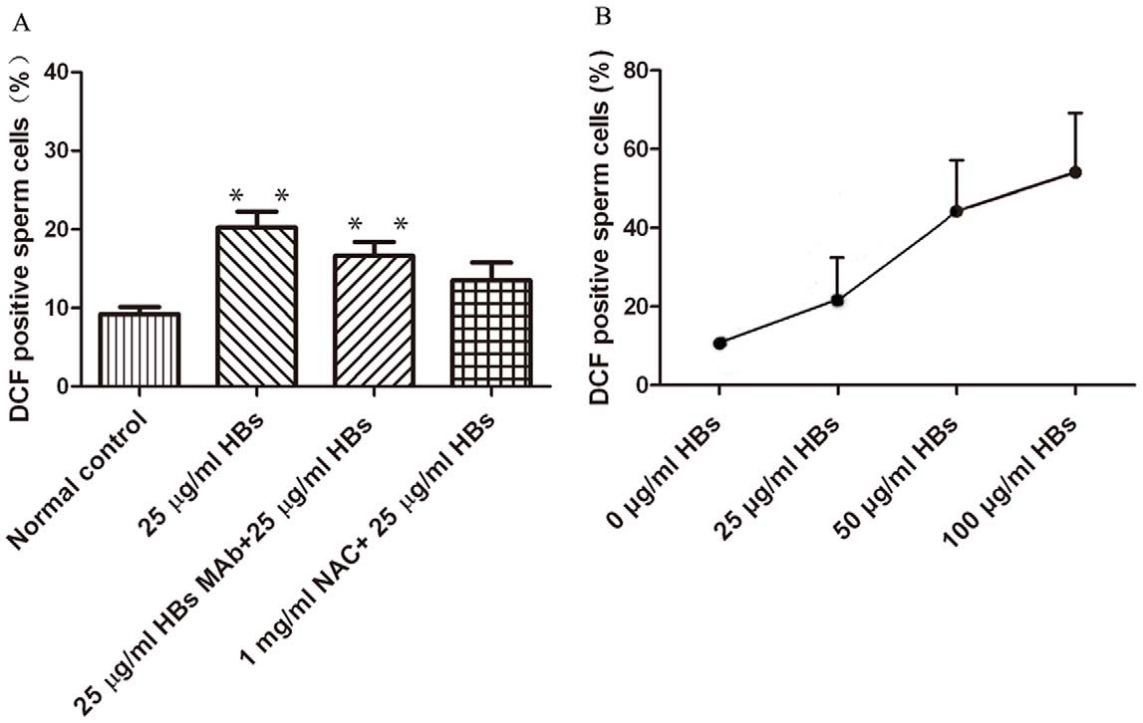
HBs-induced ROS generation in sperm cells. A: Comparison between the control, 25 µg/ml of HBs exposure, 25 µg/ml HBs MAb (pretreated)+25 µg/ml HBs exposure, and 1 mg/ml NAC (pretreated)+25 µg/ml HBs exposure(^**^ *P*<0.01). B: HBs exposure caused dose-dependent increase in ROS production in sperm cells. The data are representatives of five independent experimental replications (five individuals).

ROS levels were measured by flow cytometry using a 2′,7′-dichlorodihydrofluorescein diacetate (DCFH-DA) fluorescent probe. The results are shown in Table 1 and Figure 1. A significant increase in ROS positive cells was observed after 3 h exposure to 25 µg/ml of HBs as compared to the control. The average rate of dichlorodihydrofluorescein (DCF) positive cells was 20.25±2.04% in the exposed group, while only 9.20±0.90% in the unexposed group contributed to ROS production (P<0.01) (Fig. 1A and Table 1). The average rate of DCF positive cells in the 25 µg/ml HBs plus 25 µg/ml HBs MAb- exposed group (16.64±1.79%) was lower than that in 25 µg/ml HBs-exposed group alone (20.25±2.04%; P<0.01), which further confirmed that the increase in ROS level was caused by HBs exposure (Fig. 1A). The average rate of DCF positive cells in the group pretreated with N-Acetylcysteine (NAC) also markedly declined (P<0.01) (Fig. 1A). In sperm cells, HBs exposure increased ROS generation in a dose-dependent manner (Fig. 1B).

**Table 1:** Related parameters in the test and control groups.

Related Parameters	Average values in Controls	Average values in HBs exposure	
		(25 µg/ml)	(50 µg/ml)
DCF-positive sperm cells (%)	9.20±0.90	20.25±2.04**	
MDA (µmol/10^6^ cells)	0.074±0.20	0.58±0.21	0.85±0.27*
TAC (mM/10^6^ cells)	0.20±0.05	0.17±0.07*	
Annexin V–positive/PI-negative sperm cells (%)	1.39±0.77	5.95±1.33**	
Caspase-3 positive sperm cells (%)	47.11±4.92	54.34±3.37*	
Caspase-8 positive sperm cells (%)	39.18±3.46	56.74±2.11*	
Caspase-9 positive sperm cells (%)	27.23±3.80	45.26±3.84**	
TUNEL-positive sperm cells (%)	12.81±3.80	27.04±3.12**	

*Note:* These data are representatives of five independent experimental replications (five individuals). The average values are expressed as mean ± SEM. A paired-samples t test was conducted to evaluate the impact of HBs exposures.

* P<0.05;

** P<0.01.

### Effects of HBs on MDA levels and TAC activity in sperm cells

**Figure 2:**
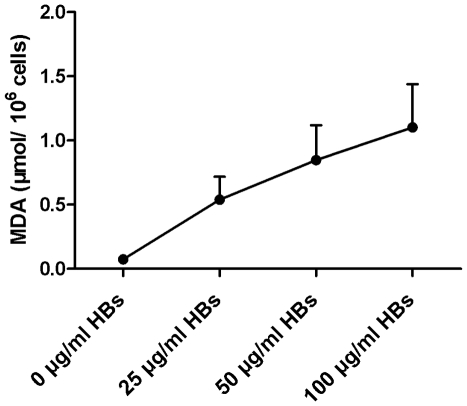
Dose-dependent effects of HBs exposure on lipid peroxidation of sperm plasma membranes. The sperm cells were exposed to HBs (0, 25, 50, 100 µg/ml) for 3 h. The MDA levels rose with increasing concentrations of HBs. The data are representatives of five independent experimental replications (five individuals).

**Figure 3:**
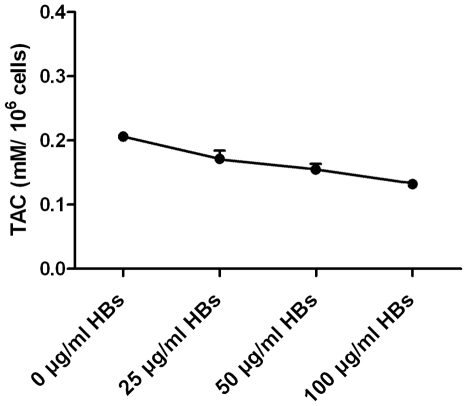
Dose-dependent effect of HBs exposure on the total antioxidant capacity in sperm cells. Sperm cells were exposed to HBs (0, 25, 50, 100 µg/ml) for 3 h. The TAC levels declined with increasing concentrations of HBs. The data are representatives of five independent experimental replications (five individuals).

Aldetect (MDA-specific) lipid peroxidation assay and ferric reducing ability of plasma (FRAP) assay were performed to determine the relationship between HBs exposure and lipid peroxidation, and total antioxidant capacity in sperm cells, respectively. As shown in Figure 2, the MDA levels rose with increasing concentrations of HBs when sperm cells were exposed to HBs (0, 25, 50, 100 µg/ml) for 3 h. In contrast, the TAC levels declined with increasing concentrations of HBs when the sperm cells were exposed to HBs (0, 25, 50, 100 µg/ml) for 3 h (Fig. 3). The sperm cells exposed to 50 µg/ml of HBs for 3 h had significantly higher level of MDA than that in the control group (0.85±0.27 µmol vs. 0.074±0.20 µmol; P<0.05), while the TAC level was positively decreased when compared with the control (0.17±0.07 mM vs. 0.20±0.05 mM; P<0.05) (Table 1).

### Externalization of PS in sperm cells induced by HBs

**Figure 4:**
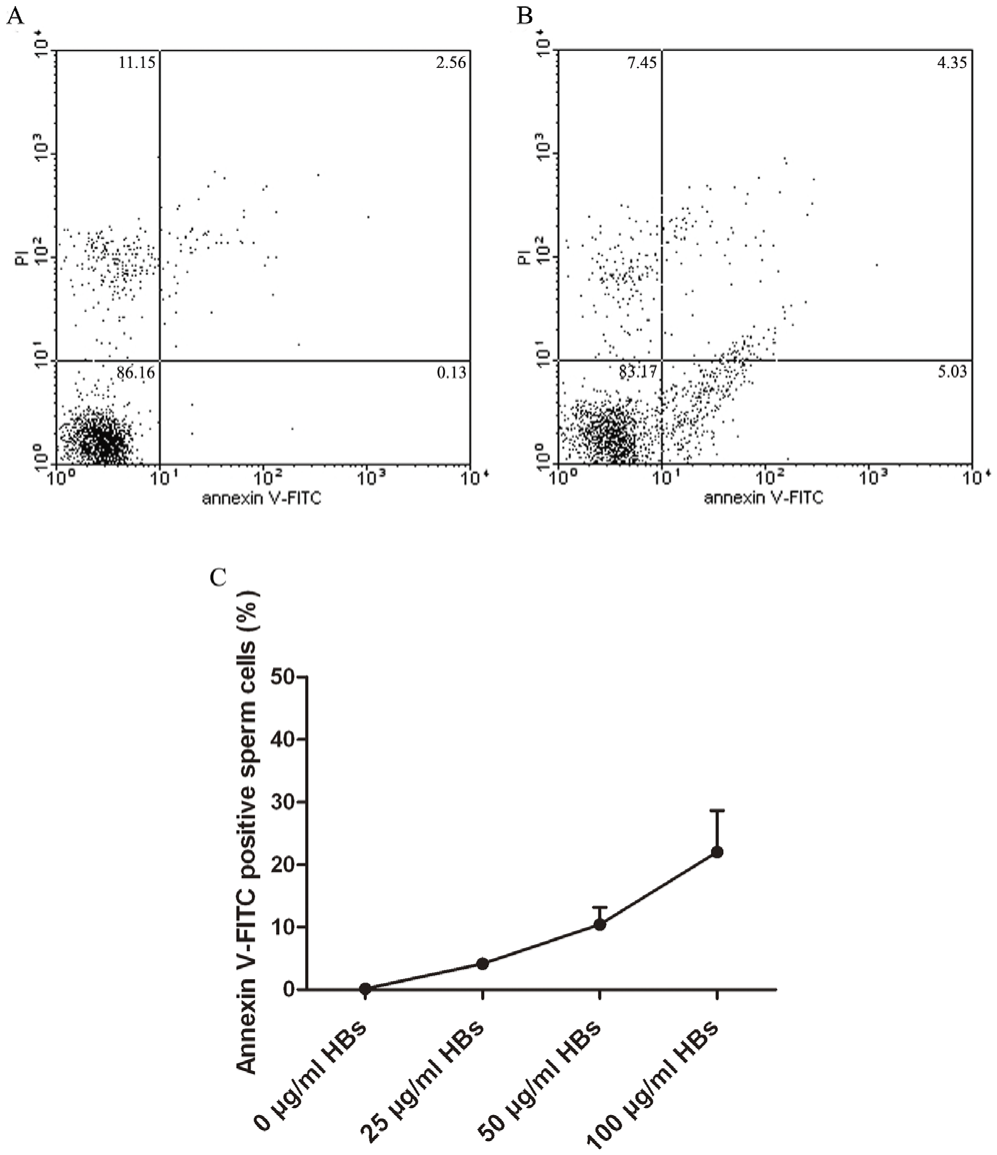
Effects of HBs on PS externalization in sperm cells. Sperm cells were unexposed (A) or exposed to 25 µg/ml HBs for 3 hours (B). Cells were stained with FITC annexin V and PI, and analyzed by flow cytometry. Unexposed cells were primarily FITC annexin V and PI negative, indicating that they were viable and not undergoing apoptosis (A). After a 3 hour exposure (B), there were primarily two populations of cells: Cells that were viable and not undergoing apoptosis (FITC annexin V and PI negative) and cells undergoing apoptosis (FITC Annexin V positive and PI negative). A minor population of cells were observed to be FITC annexin V and PI positive, indicating that they were in end stage apoptosis or already dead. The effects of HBs exposure on PS externalization in sperm cells showed dose dependence. (C).

The apoptosis-inducing effects of HBs were assessed by flow-cytomeric analysis using double staining with annexin V and PI. As seen in Figure 4, the sperm cells were either unexposed (Figure 4A) or exposed to 25 µg/ml HBs for 3 hours (Figure 4B). The effects of HBs exposure on PS externalization in sperm cells exhibited dose dependence (Figure 4C). After 3 h of exposure to HBs, the average rate of annexin V positive and PI negative cells was 5.95±1.33% (Table 1), which was significantly different from 1.39±0.77% in the control (P<0.01).

### HBs induces the activation of caspases-3, -8,- 9 in sperm cells

**Figure 5:**
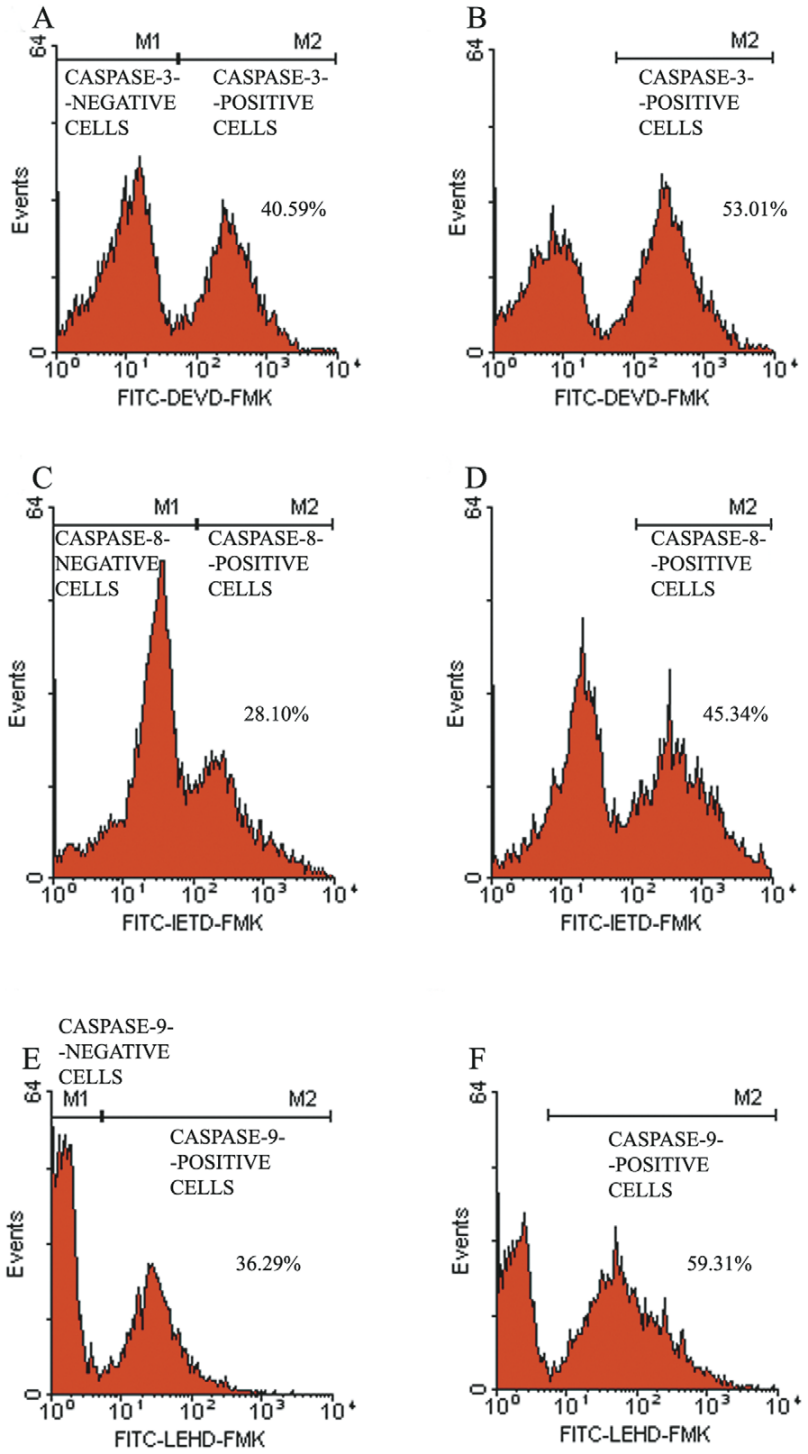
Effects of HBs exposure on caspases-3, -8, -9 activations in sperm cells. The flow cytometry frequency histograms showed the caspases-3, -8 and -9 activation distributions for the sperm cells unexposed (5A, 5C, 5E) and exposed to 25 µg/ml HBs for 3 h (5B, 5D, 5F), respectively. Cells were labeled with 1 µl of FITC-DEVD-FMK, FITC-IETD-FMK and FITC-LEHD-FMK for 1 h, respectively followed by washing and analysis by flow cytometry. All the histograms of the number of events (Y-axis) versus the fluorescence intensity (X-axis) showed two peaks. In the individual experiment, there were a markedly increase of the caspases-3, -8 and -9-positive cells (53.01%, 45.34% and 59.31%) in the exposed cells when compared with the unexposed cells (40.59%, 28.10% and 36.29%), indicating that HBs exposure was able to induce caspases-3, -8 and -9 activation in sperm cells.

**Figure 6:**
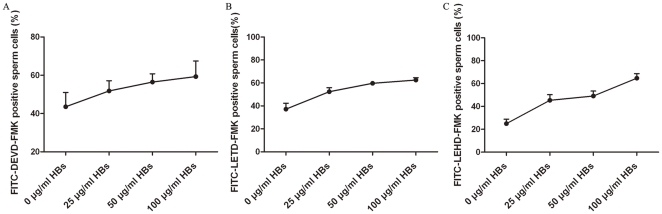
Effects of HBs exposure on caspases-3, -8, -9 activations in sperm cells. The relationships between the dose of HBs exposure and caspases-3, -8, -9 activations in sperm cells were investigated. The effects of HBs exposure on caspases-3, -8, -9 activations in sperm cells showed a manner of dose dependence. (6A, 6B, 6C).

Apoptosis is dependent on the activation of a group of proteolytic enzymes called caspases. A possible link between HBs exposure and caspases-3, -8 and -9 activations in sperm cells was investigated to determine whether HBs exposure can cause apoptosis. The sperm cells in the test and control groups were labeled with 1 µl of FITC-DEVD-FMK, FITC-IETD-FMK and FITC-LEHD-FMK for 1 h, respectively followed by washing and analysis by flow cytometry. The results showed in Table 1 and Figure 5 and 6. In the individual experiment, the average rates of caspases-3, -8 and -9 positive cells (53.01%, 45.34% and 59.31%) in the exposed groups were markedly increased (Fig. 5. B, D, F) when compared with those in the unexposed groups (40.59%, 28.10% and 36.29%) (Fig. 5. A, C, E). In five experiments, after 3 h of exposure to 25 µg/ml HBs, the average rates of caspases-3, -8 and -9 positive cells were 54.34±3.37%, 56.74±2.11% and 45.26±3.84%, respectively (Table 1), significantly higher than those (47.11±4.92%, 39.18±3.46% and 27.23±3.80%) in the controls (P<0.05), indicating that HBs exposure was able to induce caspases-3, -8 and -9 activation in sperm cells. The effects of HBs exposure on caspases-3, -8 and -9 activations in sperm cells exhibited dose dependence (6A, 6B, 6C).

### Effects of HBs exposure on oxidative DNA damage in sperm cells

**Figure 7:**
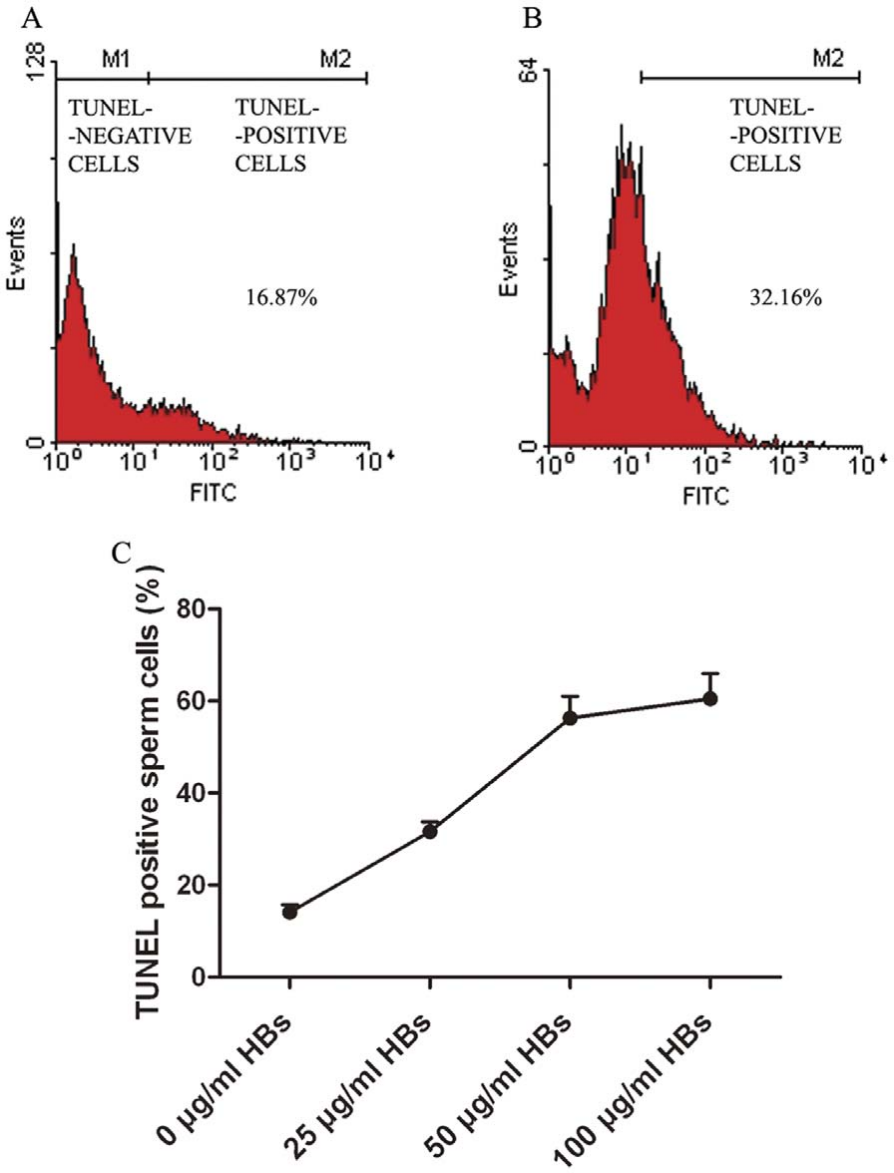
The effects of HBs exposure on oxidative DNA damage in sperm cells. HBs-induced oxidative DNA damage in sperm cells in the control group (A) and the test group (B) was assessed by flow-cytometric analysis using TUNEL assay. The rates of TUNEL-positive sperm cells with nuclear DNA strand breaks read from the M2 marker, but the cells without fragmented DNA (TUNEL-negative) from the M1 marker. The x-axis: FL1 channel - the intensity of fluorescence in the green spectrum, the y-axis: depicts the frequency in terms of the number of cells; the fluorescence intensity scale is expressed as “channel number” (0–10^4^). The effects of HBs exposure on oxidative DNA damage in sperm cells exhibited dose-dependence (C).

The effects of HBs exposure on oxidative DNA damage in sperm cells in the control and the test groups were assessed by flow-cytomeric analysis using TUNEL assay. The results are shown in Table 1 and Figure 7. After 3 h of exposure to 25 µg/ml HBs, the average rate of TUNEL positive cells was 27.04±3.12% (Table 1), which was significantly higher than that in the control (12.81±1.23%) (P<0.01). The effects of HBs exposure on oxidative DNA damage in sperm cells also demonstrated dose dependence (Fig. 7C).

## Discussion

HBV DNA presented in sperm cells was first addressed by French scientists [4] who proposed that HBV may be a cause of male infertility by damaging spermatozoa. Subsequently, it was reported that in the male genital tract, viral infections could lead to an oxidative stress by spermatozoa and leukocytes including neutrophils and macrophages and a lack of antioxidant protection [24]. Although viral infection can affect a man's fertility, however up to now, only scant information is available on the influence of HBV infection on the sperm function.

### HBs exposure induced oxidative stress in sperm cell

Successful fertilization requires a sperm plasma membrane with normal integrity and functions [25]. The numerous functions of the membrane are related to cell metabolism, for maintaining sperm motility, capacitation, acrosome reaction and sperm-oocyte interactions [26]. It has been documented that ROS plays a key role in inducing sperm damage [27]–[32]. In the present study, a significant increase in ROS positive cells was observed after 3 h exposure to 25 µg/ml of HBs when compared with control (P<0.01). Conversely, the average rates of ROS positive cells significantly reduced when sperm cells were pretreated with HBs monoclonal antibody or the ROS scavenger NAC, respectively. These data provided solid evidences that HBs exposure was able to increase ROS production in sperm cells. Furthermore, our results revealed that the exposure of sperm cells to HBs caused a dose-dependent ROS generation. In the previous study, however, Zhou et al reported that the incubation of sperm with HBs plus HBs MAb apparently accelerated the sperm motility loss [11] and showed a harmful effect on sperm functions. It seems to be conflicting with the results of the present study, in which HBs plus HBs MAb significantly decreased ROS generation in sperm cells and showed a beneficial effect on sperm functions. The reason for this discrepancy might be that HBs MAb played different roles. On one hand, HBs MAb can directly neutralize the biological activity of HBs to reduce ROS generation in sperm cells, and on the other hand, HBs MAb can bind to HBs to form HBs–HBs MAb complex that accelerated the sperm motility loss. Some also reported the similar findings in which the neutralizing effect of induced anti-HBs immunoglobulins decreased the HBsAg in the serum but the induced cellular product of HBs–HBs MAb exhibited stronger cytotoxicity to T cells from mouse spleens than that of HBs alone [33]. It suggested that the mechanism in adverse effects caused by HBs–HBs MAb complex might be different from the mechanism by which HBs MAb neutralized the biological activity of HBs in sperm cells.

Lipid peroxidation of the sperm membrane can result in the loss of membrane integrity including sperm-oocyte fusion and the ability to undergo acrosomal exocytosis and increase in membrane permeability, causing a loss of capacity to regulate the intracellular concentrations of ions involved in the sperm movement [34]. In the present study, the sperm cells exposed to 50 µg/ml of HBs for 3 h had significantly higher level of MDA than that in the control group (P<0.05). The MDA levels rose with increasing concentrations of HBs. These suggested that HBs exposure was able to induce lipid peroxidation in sperm cells.

There are, however, many antioxidants in the body which deactivate free radicals and act as inhibitors of the oxidation process, even at relatively low concentrations and thus have diverse physiological roles. What are the effects of HBs on antioxidants in sperm cells? In the present study, the TAC of sperm cells was investigated and the results showed that TAC levels in sperm cells exposed to HBs declined with increasing concentrations of HBs. It indicated that HBs exposure was not only able to induce ROS generation and lipid peroxidation but also reduce antioxidant capacity in sperm cells, resulting in oxidative stress, an imbalance between the production and manifestation of ROS and a biological system's ability to readily detoxify the reactive intermediates, leading to sperm dysfunctions.

### HBs exposure induced apoptosis in sperm cell

It is well known that apoptosis plays a critical role in many physiological and pathological processes [35]. An altered apoptosis process has been found to be closely associated with male infertility and with sperm quality such as motility, viability and sperm defects [36], [37]. In the present study, the relationship between HBs exposure and sperm apoptosis was investigate by using annexin-V staining for detection of membrane PS externalization, TUNEL for the measurement of DNA fragmentation and fluorochrome-labeled caspase inhibitors for detection of caspases activation. In the present study, after 3 h of exposure to 25 µg/ml of HBs, the average rate of annexin V positive and PI negative cells was significantly different as compared to the control (P<0.01). In addition, the effects of HBs exposure on PS externalization in sperm cells showed dose dependence. It suggested that HBs exposure was able to induce apoptosis in sperm cells. The caspase activity is also an early indicator of apoptosis. The caspases are a family of proteins within the cell as inactive pro-forms which can be cleaved to form active enzymes following the induction of apoptosis. In the present study, a possible link between HBs exposure and caspases-3, -8 and -9 activations in sperm cells was investigated. The results showed that there were a markedly increase of caspases-3, -8 and -9 positive cells in the exposed cells when compared with the unexposed cells, indicating that HBs exposure was able to induce caspases-3, -8 and -9 activation in sperm cells. The effects of HBs exposure on caspases-3, -8 and -9 activations in sperm cells also exhibited dose dependence. DNA fragmentation is commonly considered as the key feature of apoptosis in many cell types. In the present study, we detected apoptosis in sperm cells after 3 h of exposure to 25 µg/ml of HBs, the average rate of TUNEL positive cells was significantly elevated as compared to the control (P<0.01). In addition, the effects of HBs exposure on oxidative DNA damage in sperm cells also showed dose dependence.

### Oxidative stress, loss of MMP and apoptosis caused sperm dysfunctions

Since the mitochondria of the sperm midpiece generate energy to support motility, the state of sperm MMP is a useful indicator of functional impairments on the reproductive system [38]. It was well documented that a high correlation between poor sperm mitochondrial functions and diminished motility and reduced fertility [39]–[41]. In our previous study, it was detected that HBs caused sperm MMP loss to reduce sperm motility, leading to the death of sperm cells and a lower fertilization rate and a lower fertilizing index [11]. The present study showed that HBs exposure was not only able to induce ROS generation and lipid peroxidation but also cause apoptosis in sperm cells, which were intimately associated with the findings in the previous study. It has been confirmed that the increased ROS generation can cause mitochondrial injury with a marked decrease in MMP [41]. Damaged mitochondria may release some pro-apoptotic molecules including cytochrome *c* and apoptosis-inducing factor, which caused caspase-dependant and caspase-independent apoptosis, respectively [42]. The loss of MMP could lead to lack of energy required for sperm motility, and lipid peroxidation of the sperm membrane could result in the loss of membrane integrity, and the pathway(s) of sperm cell death might be switched from caspase-dependent to caspase-independent apoptosis [42]. All these events can ultimately affect sperm functions or result in the death of sperm cells, leading to loss of sperm motility and reduced fertilizing ability.

### Oxidative stress caused DNA damage and sperm chromosome aberrations

Oxidative stress as one of the major caused of sperm DNA damage has been widely adopted. This kind of damage is characterized by single and double DNA strand breaks. Although DNA damage of the first type may be repaired by the oocyte or the embryo, fertilization of an oocyte by a spermatozoon with extensive double-stranded DNA fragmentation is virtually not repairable and incompatible with normal embryo and fetal development [43]. The double-stranded DNA breaks may cause chromosomal aberrations. It was reported by our laboratory that the higher frequency and various types of chromosome aberrations were observed in sperm cells from the patients with HBV chronic infection [5]. These events were probably caused by the imbalance between ROS production and TAC induced by HBs, leading to the accumulation of the unrepaired DNA damage and formation of sperm chromosome aberrations.

Taken together, our data provided the solid evidence that HBs exposure could cause a series of deleterious events in sperm cells such as induction of ROS generation and lipid peroxidation, reduction of total antioxidant capacity, PS externalization, activation of caspases, and DNA fragmentation, resulting in increased apoptosis of sperm cells, the loss of sperm membrane integrity and sperm dysfunctions.

## Materials and Methods

### Ethical approval

After the informed consent approval was obtained, the human sperm samples were collected from the healthy male donors who were explicitly informed about the research aims, their rights and interests in the research. All the protocols used in the present study were approved by Institutional Ethical Review Board (IERB) of Shantou University Medical College (SUMC), and conformed to the ethical guidelines of the 2008 Declaration of Helsinki as reflected in a prior approval by the institution's human research committee.

### Preparations of human spermatozoa

Human sperm samples were obtained by masturbation after 3 days of sexual abstinence from the healthy men. Semen samples were kept in a CO_2_ incubator (37°C, 5% CO_2_) for 30 min to allow liquefaction. Motile spermatozoa were selected by the swim-up method as follows: in each test tube, the 0.5 ml liquefied semen sample layered gently under 2 ml of biggers-whittem-whittingham (BWW) medium containing 0.3% bovine serum albumin (BSA) and incubated at 37°C in a 5% CO_2_ incubator for 1 h. The supernatant collected from tubes was centrifuged at 300×g for 5 min, and the pellet of motile sperm was washed once. The final concentration of spermatozoa was roughly adjusted to 1×10^6^ sperm/ml in BWW medium with 0.3% BSA for the following use.

### The exposure of sperm cells to HBs

The sperm cells (1×10^6^/ml) were incubated in BWW medium with various concentrations of HBs (0, 25, 50, 100 µg/ml) (NCPC GencTech Biotechnology Co., LTD., Hebei, China) in a CO_2_ incubator (37°C, 5% CO_2_) for 3 h to determine the effects of HBs exposure on sperm membrane integrity and functions. The sperm cells exposed to HBs were used as the test group, and the unexposed sperm cells were used as the control group in the following study.

### Estimation of ROS in sperm cells

The intracellular ROS level in sperm cells was measured using a ROS assay kit (Beyotime Biotech, Haimen, China). Briefly, the enriched sperm cells (1×10^6^/ml) were divided into six groups: four groups were exposed to 0, 25, 50, 100 µg/ml HBs for 3 h, respectively, and two groups were pretreated with HBs MAb (25 µg/ml) and NAC (1 mg/ml) for 30 min followed by exposure to 25 µg/ml HBs for 3 h, respectively. After removing the supernatant, the sperm cells were washed with phosphate buffered saline (PBS) and incubated with DCFH-DA at a final concentration of 10 µM at 37°C in the dark for 20 min. Then sperm cells were centrifuged and washed three times with PBS. The labeled sperm cells were analyzed by flow cytometry. The above experiment was repeated five times.

### Estimation of lipid peroxidation in sperm cells

Aldetect Lipid Peroxidation assay was used to measure LP in sperm cells. Sperm cells (1×10^6^/ml) in the test and control groups were lysed with Western and immunol precipitation lysis buffer (Beyotime Biotech, Haimen, China), respectively. The lysates were homogenized, and the homogenates were centrifuged at 1,600×g at 4°C for 10 min. The supernatants were collected and determined with Lipid Peroxidantion MDA Assay Kit (Beyotime Biotech, Haimen, China). A 200 µl of thiobarbituric acid (TBA) reagent was added to 100 µl of the sperm suspension. The mixture was treated in a boiling water bath for 15 min. After cooling, the suspension was centrifuged (1,000×g; 10 min) and the supernatant was separated, then the absorbance was measured at 530 nm and expressed as units (U) per 1×10^6^/ml sperm cells for MDA. The above experiment was repeated five times.

### Assessment of antioxidant status in sperm cells

FRAP assay was performed to measure the TAC of sperm cells in the test and control groups using a commercially available assay kit (Beyotime Biotech, Haimen, China) in a Multimode Microplate Reader (Infinite M1000, Tecan Co., Austria) according to the manufacturer's instructions. Briefly, the sperm cells were homogenized in 200 µl of phosphate buffer (pH 7.4) and sonicated over ice, and then centrifuged at 12,000×g at 4°C for 5 min to collect the supernatant for assay of TAC. The values were calculated using optical density at 593 nm and expressed as units (U) per 1×10^6^/ml sperm cells for TAC. The above experiment was repeated five times.

### Evaluation of PS externalization in sperm cells

The annexin-V–FITC Apoptosis Detection Kit (CalBiochem, CA, USA) was used to detect PS translocation from the inner to the outer leaflet of the sperm plasma membrane. The assay was carried out according to the instructions of the manufacturer. Briefly, for each assay, 1×10^6^/ml washed sperm cells in the test and control groups were gently resuspended in 0.5 ml cold PBS followed by incubating with annexin V-FITC and PI at room temperature in the dark for 15 min. To prepare the samples for flow cytometry, sperm cells were washed twice with annexin binding buffer. For statistical analysis, the cell population (FITC annexin V-positive and PI-negative) was considered positive for PS externalisation. The above experiment was repeated five times.

### Detection of active caspases-3, -8, -9 in sperm cells

The caspase activities in sperm cells were assayed using the Colorimetric Caspases-3, -8, -9 Assay Kit (CalBiochem, CA, USA), which utilize potent caspase inhibitors, DEVD-FMK, IETD-FMK and LEHD-FMK, that are conjugated to FITC as fluorescence *in situ* markers. There were three control groups in this experiment, including the inhibitors as the appropriate controls that are provided with the Kit, the culture without induction, and the culture exposed to caspase inhibitor Z-VAD-FMK at a final concentration of 1 µl/ml to inhibit caspase activation. All the test and control groups were incubated with 1 µl of the fluorescent maker in a CO_2_ incubator (37°C, 5% CO_2_) for 1 h, and subsequently washed twice with the rinse buffer on ice. Analyze samples by flow cytometry. The above experiment was repeated five times.

### Determination of DNA fragmentation in sperm cells

The FragEL™ DNA Fragmentation Detection assay kit (CalBiochem, CA, USA) was used to investigate the impact of HBs exposure on nuclear apoptosis in sperm cells according to the manufacturer's protocol with some slight modifications. Briefly, the washed sperm cells (1×10^6^/ml) in the test and control groups were fixed with 4% formaldehyde-PBS (Sigma-Aldrich, Shanghai, China) at room temperature for 30 min. Then the cells were washed once with 1 ml of PBS followed by permeabilization with 100 µl of 20 µg/ml proteinase K at room temperature for 5 min. After washing with equilibration buffer, the labeling reaction was performed by incubating cells with 60 µl of terminal deoxynucleotidyl transferase (TdT) labeling reaction mixture at 37°C for 1.5 h in the dark. TdT enzyme was not added to the negative control. The positive control was obtained by incubating one sample with 10 mg/ml DNAse at room temperature for 10 min. After labeling, the samples were washed twice with Tris-buffered saline (TBS) and analyzed with a flow cytometer equipped with a 488 nm argon-ion laser source. The above experiment was repeated five times.

### Analysis of Flow cytometry

All flow cytometric analyses were performed using a FACScan FlowCytometer (BD Biosciences, San Diego, CA). Cells were isolated from fragments by gating on the forward and side scatter signals, and then cells were detected and analyzed according to their relative fluorescence intensities compared with unstained cells. A minimum of 10,000 events were acquired and analyzed in each sample at the rate of 50–500 events per second, and data analysis was performed using BD Cell Quest and WinMDI 2.9 software. Different sperm suspensions were prepared for instrumental setting and data analysis: (1) by omitting all fluorochromes (non-specific fluorescence sample); (2) by adding only one fluorochrome (samples for compensation). Fluorescence was detected by using the FL1 channel at 488/525 nm excitation/emission for DCFH-DA, AnnexinV-FITC, FITC-DEVD-FMK, FITC-IETD-FMK and FITC-LEHD-FMK, and using the FL2 channel at 488/620 nm excitation/emission for PI.

### Statistical analysis

Data were presented as mean values ± SEM. SPSS 17.0 programs were used in the statistical analysis. A paired-samples T test was used to determine whether there is a significant difference between the average values of the test group and the control group. P-value of less than 0.05 was considered to be significant.
